# What does cannabis do to the brain before birth?

**DOI:** 10.7554/eLife.41229

**Published:** 2018-09-28

**Authors:** Stefano Musardo, Camilla Bellone

**Affiliations:** Department of Basic NeuroscienceUniversity of GenevaGenevaSwitzerland

**Keywords:** cannabis, in-utero, sex difference, social behavior, prefrontal cortex, plasticity, Rat

## Abstract

Being exposed to cannabinoids in the womb has different consequences for male and female rats.

**Related research article** Bara A, Manduca A, Bernabeu A, Borsoi M, Serviado M, Lassalle O, Murphy MN, Wager-Miller J, Mackie K, Pelissier-Alicot AL, Trezza V, Manzoni OJ. 2018. Sex-dependent effects of in utero cannabinoid exposure on cortical function. *eLife* **7**:e36234. doi: 10.7554/eLife.36234

According to the United Nations Office of Drugs and Crime, a total of 246 million people between the ages of 15 and 64 have used an illicit drug. Cannabis is by far the most widely cultivated, trafficked and used psychoactive substance, and its consumption has grown more rapidly than both cocaine and opiate abuse over the past decade. The use of marijuana during pregnancy ranges from 2% to 5%, and it increases to between 15% and 28% among young and socioeconomically disadvantaged women; many pregnant users also believe that the drug is safe with no major effects on the fetus (Committee on Obstetric Practice, 2017). The main component in marijuana, delta 9-tetrahydrocannabinol (THC), is able to cross the placental barrier and enter the fetal bloodstream, so thousands of infants are exposed to cannabis before birth. This means that the consumption of the drug during pregnancy is a major public health concern.

Although children with a history of cannabis exposure in utero have normal intelligence scores, they show deficits in attention, memory, concentration and inhibitory control, as well as high levels of depression and anxiety (Wu et al., 2011; Gunn et al., 2015; Higuera-Matas et al., 2015). Thus, it is critical to understand how THC and other cannabinoids affect a developing fetus. In the brain, THC principally targets a receptor called CB1R (short for cannabinoid receptor type 1), which is a crucial part of the endocannabinoid system – a biological network that controls a range of neuronal processes such as proliferation, migration, morphogenesis and synaptogenesis (Harkany et al., 2008; Maccarrone et al., 2014). Consequently, chronic exposure to substances that activate CB1R, like THC, can over-stimulate the endocannabinoid system and dramatically alter brain maturation, potentially causing long-lasting neurobiological changes (Renard et al., 2014). To date most studies in this field have left out female progeny, possibly because the hormonal fluctuations associated with the estrus cycle can introduce ‘noise’ in the data. However, only examining male offspring leaves a large gap in our knowledge about the effect of THC on the developing brain.

Now, in eLife, Olivier Manzoni and colleagues – including Anissa Bara and Antonia Manduca as joint first authors – report that adult male and female rats are differently affected by prenatal exposure to cannabinoids (Bara et al., 2018). The team focused on the rats’ behavior, and on the synaptic functions of their medial prefrontal cortex; together with the nucleus accumbens, this region of the brain is implicated in a number of neuropsychiatric disorders and plays a crucial role in social behaviors.

Bara et al. found that prenatal cannabinoid exposure led to a decrease of social interactions and a reduction in play behavior in adult males, but not in adult females. However, it had no impact on aggressive behaviors, suggesting that only specific aspects of social interactions are affected by exposure to cannabinoids in the womb.

Behaviors that are related to rewards are modulated by the glutamatergic circuit, which involves the prefrontal cortex and the nucleus accumbens. Focusing on this circuit, Bara et al. found that, in adult males, exposure to cannabinoids in the womb alters a type of synaptic plasticity called long-term depression in the prefrontal cortex. This plasticity was preserved in adult females, and in the nucleus accumbens in either sex. In addition, the team found that prenatal cannabinoid exposure led to an increased excitability of certain pyramidal neurons in the prefrontal cortex in male progeny only. These results strengthen the idea that cannabinoid exposure in utero has a different effect on the two sexes (Figure 1).

**Figure 1.**
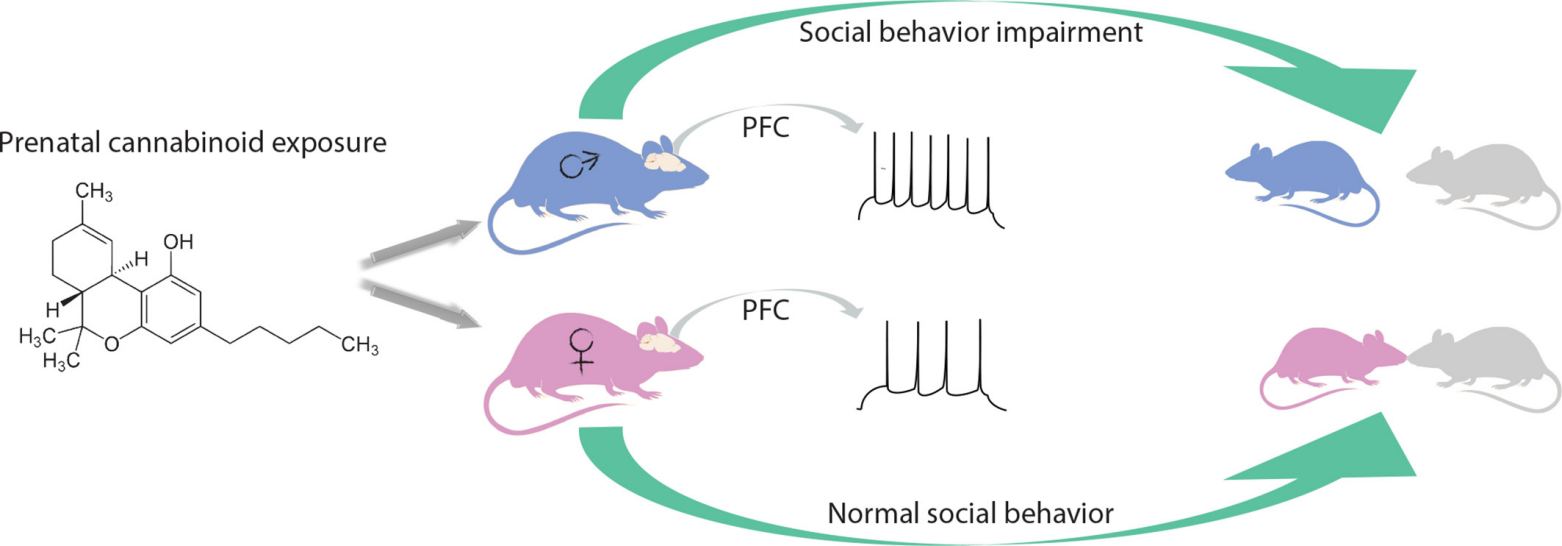
Effects of prenatal exposure to cannabinoids on male and female rats. Being in contact with cannabinoids in the womb leads to changes in the medial prefrontal cortex (PFC) of adult male rats (blue), for example causing prefrontal cortex pyramidal neurons to fire more easily (spikes). These animals interact less with their peers and do not play as much. Female rats (pink) with the same life history are not affected by prenatal cannabinoid exposure.

In order to identify a possible strategy to rescue the effects of THC, the team analyzed how prenatal cannabinoid exposure affects the expression of key components of the endocannabinoid system. In male offspring, Bara et al. observed decreased levels of mRNA for the brain receptor mGlu5; in female progeny they detected lower levels of mRNA for mGlu5, but also for a receptor called TRPV1 and an enzyme, DAGLα. Considering these results, the group decided to amplify the level of mGlu5 signaling by using drugs known as positive allosteric modulators, which fully restored the ability of excitatory medial prefrontal cortex synapses to express long-term depression. Systemic administration of the compound was able to normalize social interaction in male rats exposed to cannabinoids in the womb.

When investigating how the positive allosteric modulator of mGluR5 works, Bara et al. – who are based at Aix-Marseille University, the APHM hospital in Marseille, Indiana University and the University of Rome Tre – observed that the drug engaged both CB1R and TRPV1. In naïve rats, long-term depression is inhibited by CB1R antagonists in males, but by TRPV1 antagonists in females. These data suggest that TRPV1, rather than CB1R, mediates synaptic plasticity in female rats exposed to cannabinoids before birth. Based on these findings, the researchers tried to rescue the effects of prenatal cannabinoid exposure with a molecule that increases the levels of one of TRPV1’s ligands and enhances the activity of the receptor. In male progeny, the drug corrected both the synaptic and behavioral deficits associated with cannabinoid.

The prefrontal cortex is a critical neuroanatomical hub that projects neurons to regions of the brain that are involved in reward and social behaviors (Otis et al., 2017). These downstream circuits may be impaired during early exposure to cannabinoids, as suggested by the deficits in social interactions of male rats. It will be interesting to understand why and how prenatal exposure to cannabinoids affects these circuits in both sexes.
